# Thoracoplasty Without Rib Resection by the Sawamura Technique: A Forgotten Technique for Effective Complex Pleural Empyema Management in a Single-Step

**DOI:** 10.3390/jcm14217673

**Published:** 2025-10-29

**Authors:** Kostas Kostopanagiotou, Kostas Papagiannopoulos, Jacek Szulc, Norbert Wójcik, Małgorzata Edyta Wojtyś

**Affiliations:** 1 Thoracic Surgery Department, Attikon University Hospital of Athens, 12462 Athens, Greece; kostop@hotmail.co.uk; 2 Thoracic Surgery Department, St. James’s University Hospital, Leeds LS9 7TF, UK; 3 Department of Thoracic Surgery and Transplantation, Pomeranian Medical University in Szczecin, Alfreda Sokołowskiego 11, 70-891 Szczecin, Poland; 4 Clinic of General, Minimally Invasive and Gastroenterological Surgery, Pomeranian Medical University in Szczecin, Unii Lubelskiej 1, 71-252 Szczecin, Poland

**Keywords:** chest wall, thoracoplasty, empyema, pleural space

## Abstract

Treatment for complex pleural empyema often requires thoracoplasty with rib(s) resection to remodel the thoracic cage and obliterate chronic infected pleural cavities. Such procedures are complicated and result in permanent body deformation, which is not acceptable by most adults. Standard decortication often fails as there is residual space for reinfection development, and often necrotizing pneumonia co-exists. Here we describe the surgical management of three complicated adult patients using the modified version of the Sawamura technique which involves debridement and partial decortication followed by ribs stripped of periosteum and surrounding soft tissues, to allow collapse deep into the pleural cavity, thereby obliterating the chronic space in conjunction with partial lung re-expansion. We utilized the serratus muscular flap to repair any bronchial defects due to resected gangrenous parenchyma. No further reoperations were necessary, and no residual effusions were drained. At the 6-month follow-up, these three patients experienced no complications, and their body shapes and functionality were unaltered. This modified Sawamura technique offers an effective single-step treatment while being cosmetically suitable for young adults, and presents an extremely attractive option in countries with limited healthcare resources.

## 1. Introduction

**Figure 1:**
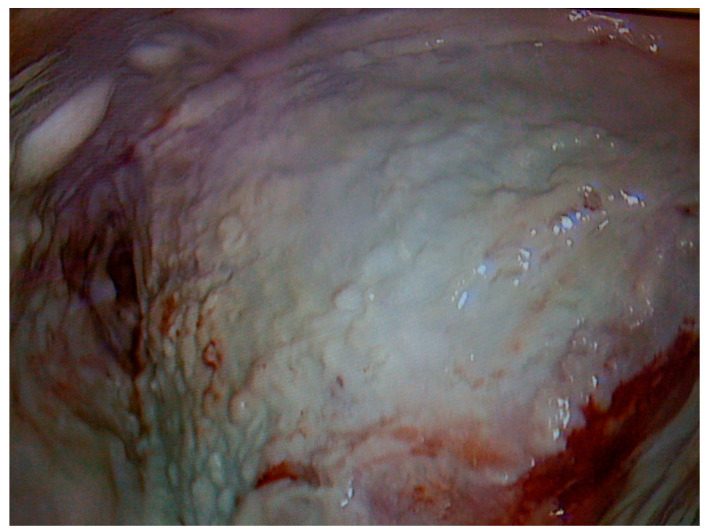
Intra-operative view of the pleural cavity in a complex pleural empyema case, resulting from failed medical thoracoscopy and talc pleurodesis. The cavity is filled with pus, and the lung is trapped, preventing re-expansion. Decortication often fails if there is underlying parenchymal inflammation, or even worse, parenchymal necrosis.

Complex infected pleural spaces are difficult to manage, especially after previous failed medical, interventional, or surgical attempts. Combined culture-driven antibiotic regimens and repetitive tube thoracostomy are frequently unsuccessful for treating compartmentalized cavities filled with fibrinopurulent material and trapped lung as shown in Figure 1 [1,2,3,4]. Prolonged hospital admissions and repetitive failed interventions means that complex pleural empyema remains a challenge for healthcare professionals with a high in hospital mortality rate, which was reported by Perez-de-Paz to be 11% [5]. Surgery involving debridement and washout is indicated for infection source control and decortication for lung re-expansion; however, these targets are not always attained. In the pre-minimally invasive surgery era, popular techniques used in the treatment of complex pleural infections with septic chronic spaces included long-term drainage and thoracotomy procedures combined with Eloesser flap thoracostomy, extensive rib thoracoplasty, Clagett procedure and even costopleuropneumonectomy [6]. Currently, video-assisted thoracoscopic surgery (VATS) for decortication is the mainstay of treatment, but the success strongly depends on whether the lung can be adequately decorticated and re-expanded [7,8]. Failure can be related to poor lung compliance, inability to decorticate the lung, underlying parenchymal active infection and residual space with a large disproportion of ratio of the lung volume to the pleural cavity, which may necessitate collapse strategies [9,10]. For the majority of modern thoracic surgeons, especially among those lacking extensive experience in this type of surgery, a failed decortication, either open or VATS, equals deforming thoracoplastic surgery. However, reviewing traditional approaches resurfaces the forgotten Sawamura technique, an effective but largely disused open thoracotomy procedure, which can be combined with other techniques to control challenging pleural infections without deforming steps or extensive decortications and in its principle, it refers to soft elements collapse initially used in the treatment of pleural tuberculosis. Although tuberculosis remains rare in most developed countries, complex pleural infections can occur in immunocompromised adults and sadly in mismanaged medical cases [11]. Below we present three representative cases where the Sawamura thoracoplasty was the definite effective procedure.

## 2. Technique Description

### 2.1. Procedure Planning

Candidates for the procedure include those resistant to medical treatment(s) and successive failed percutaneous interventions that develop infection chronicity in their parenchyma and pleural cavity with trapped (and potentially necrotic) lung. Physical fitness is inevitably reduced in septic patients; however, patients must have adequate stamina to withstand general anesthesia and thoracotomy. There are no rules for preoperative evaluation as these patients cannot be accurately evaluated with spirometry; however, we suggest an evaluation of the cardiovascular risk at least with physical examination, detailed history regarding cardio-pulmonary disease and echocardiography as minimum. Recent imaging of the chest by computed tomography (CT-scan) with intravenous contrast should always be available by the referring medical team. These should be studied thoroughly to evaluate (a) the thoracic cage anatomy, (b) the parenchymal condition (i.e., healthy, atelectatic, consolidated, gangrenous-necrotic), (c) the location and extent of the pleural cavity space with designation of the most gravity-dependent drainage area in case only a thoracostomy is performed, and (d) the thickness of the soft elements of the chest. We do not advise only regular chest x-rays, unless the local resources are limited, as these can only provide details about the chest morphology but limited details on the parenchyma. Magnetic resonance imaging is of little value as all of the desired information can be equally well provided by a CT scan. As decortications and chest wall resections can lead to significant blood loss, it is always advisable to optimize the pre-operative hemoglobin levels and have cross-matched red blood cells for intraoperative transfusion. The availability of a high-dependency (HDU) or intensive care unit (ICU) bed is desirable considering the septic profile and severity of the thoracotomy procedure; however, this procedure is addressed mainly for the areas with limited resources and preoperative preparation, and optimization of the patient should exclude this need.

### 2.2. Operative Technique

**Figure 2:**
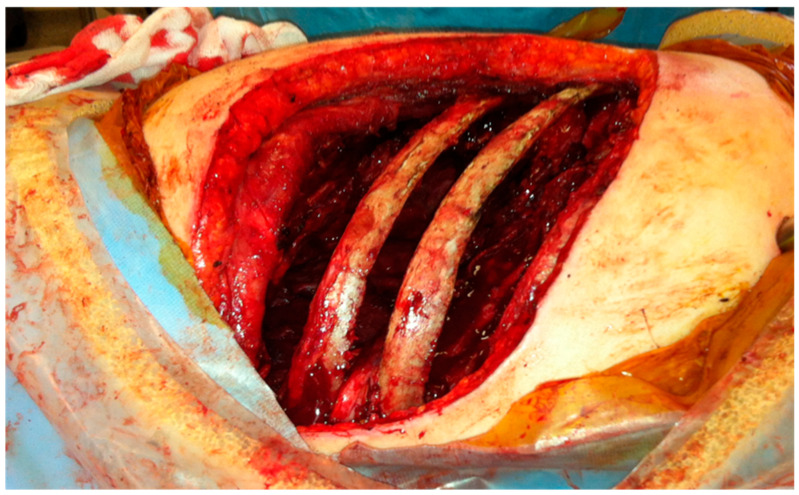
Intraoperative picture of a patient treated with Sawamura thoracoplasty; ribs 5, 6 and partially the 7th are stripped of soft tissues, allowing free collapse in the pleural cavity.

This is a strictly open procedure by tailored thoracotomy with the patient in a lateral thoracotomy position under general anesthesia and double lumen endotracheal ventilation of a single lung. Unless performed urgently, we strongly advise single lung ventilation with contralateral bronchial blockage to avoid the spillage of septic material in the healthy lung. The use of epidural anesthesia is advised if resources permit that. On the contrary, paravertebral blocks are of no value due to the extensive pleural mobilization in this procedure. Thoracotomy is designed to match the deepest part of the underlying cavity and long enough to permit pleural decortication. In case the Sawamura technique is not possible, then this thoracotomy should permit the formation of an Eloesser flap. As described below in our series, the typical or atypical resection of the lung is always a (feared) additional challenging step, thus the incision should permit hilar and fissural dissection of the central broncho-vascular structures. At thoracotomy initiation the serratus anterior and latissimus dorsi muscles should be preserved for potential flap use as described in the below cases. The next step is the completion of the thoracotomy by entering the pleura cavity, debridement and initiation of the decortication of the visceral pleura. Tissue and fluid cultures should be obtained at this step in order to direct future antimicrobial decisions. If there is parenchymal destruction, then this should be addressed, i.e., typical (lobectomy) or atypical wedge resection for abscess or necrosis. The presence of bronchopleural fistula should be investigated and accordingly an attempt should be made to close or repair it by obliteration with a muscle flap as described below. Visceral decortication should be commenced to the extent possible, unless the parenchyma is damaged to an extent prohibiting decortication. At that point, the decision for decortication only, an Eloesser window or Sawamura should be undertaken. If the lung remains unexpanded under manual (bag) ventilation by the anesthetist and if there are substantial soft tissues at the chest wall, the Sawamura thoracoplasty can be attempted. The ribs above and below the thoracotomy level are stripped from all muscular and fascial elements using gentle diathermy without creating defects on the surface. Figure 2 shows an intraoperative view with ribs 5 and 6 stripped off and soft tissues collapsing in the pleural cavity. This muscular covering layer which includes the neurovascular intercostal bundles should be intact to seal the cavity. The number of ribs to be stripped is selected according to the cavity size. Two ribs above and two ribs below are sufficient. After completing all intrathoracic maneuvers, the thoracotomy is closed meticulously with absorbable thick running Vicryl 0 or 2-0 suture after inserting one or two wide (28-36Fr) chest drains. The stripped ribs are in place, preserving the normal shape. The extrathoracic pericostal space is drained by a vacuum Redon-type narrow tube connected to a drainage-bottle. The thoracotomy incision is then closed as per standard.

### 2.3. Postoperative Considerations

As for typical thoracotomy adequate analgesia, respiratory physiotherapy, early physiotherapy and hemorrhage monitoring are mandatory. Chest drains should not remain for long and ideally if there is no air leak, these should be removed early to allow physiotherapy and early discharge. Antibiotics should be continued according to infectiology experts and only upon indications. Most patients will have resistant strains that may require long regimens in which case oral medications should be preferred over intravenous to allow early discharge. Regular follow-up with chest x-rays should be sufficient, whereas CT-scans are preferred when there are concerns of recurrent pneumonia.

## 3. Case Descriptions

### 3.1. Patient 1

A 60-year-old male obese heavy smoker developed left main pulmonary artery embolism, initially diagnosed as left lower lobar pneumonia and treated with antibiotics until acute respiratory failure developed, and he was admitted to the intensive care unit (ICU). Four weeks after admission and while requiring constant support from mechanical ventilation under tracheostomy, he developed left lower lobe necrosis with a bronchopleural fistula and massive air leakage from the previously inserted chest tube for parapneumonic effusion, resulting in rapid desaturation and gross loss of delivered tidal volume. On an urgent basis, the patient underwent exploratory left posterolateral thoracotomy under double-sided ventilation. The necrotic lower lobe was removed with atypical lobectomy, and the bronchial stump was obliterated with a Serratus anterior flap sutured on the bronchial open stump, using interrupted prolene 3-0 sutures. Decortication of the pleural cavity was impossible because of the unknown state of the underlying fragile parenchyma; thus, in addition to the lobectomy, the upper lobe remained trapped. Moreover, the patient had an increased risk for operative bleeding due to the ongoing effect of a therapeutic heparin regimen. To obliterate the pleural cavity, the soft-tissue elements of the left lateral chest wall, including the thick inflammatory parietal pleura and intercostal muscles, were dissected from the ribs and then were sutured as a mobile layer on the visceral pleura of the upper lobe, thereby obliterating the basal space. Two 36Fr intercostal chest drains were basally inserted, and a single chest drain was inserted in the subcutaneous space, all on suction until removal. At the end of the procedure, there was no air leakage, and the patient restored acceptable ventilation until extubation one week later. He remained on heparin for 6 more months, succeeding in full physical recovery without thoracoplastic deformation.

### 3.2. Patient 2

A 75-year-old male cachectic smoker of low socio-economic status underwent improperly treated antibiotic-resistant right-sided pneumonia with parapneumonic effusion due to Gram-positive cocci in pleural fluid. He underwent several unsuccessful pleural drainage procedures, including small-bore Seldinger type tubes and twice closed tube thoracostomy, failing to re-expand the right trapped lung and control the pleural infection.

**Figure 3:**
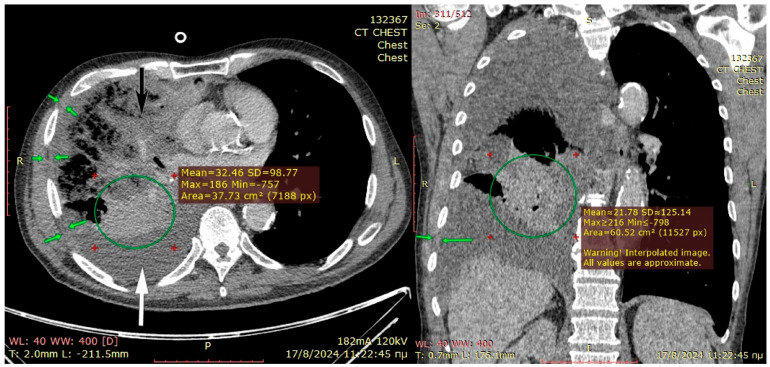
Preoperative planning CT-scan for Case 2 where the severely infiltrated middle lobe (black arrow) mandates salvage lobectomy to avoid gangrenous conversion and sepsis. The lower right lobe (green circle) is grossly atelectatic, but assessing its viability and attempting decortication is only possible through thoracotomy. The green arrows in both pictures show a moderate chest wall thickness. The decision for Sawamura can only be undertaken intraoperatively. An open thoracostomy window could also be offered to this patient if only the community can offer daily dressing changes.

The preoperative CT scan is shown in Figure 3. The patient became septic and developed middle-lobe necrosis, for which he underwent an acute open lobectomy combined with an unsuccessful decortication of the remaining two lobes due to emphysematous frail parenchyma and post-operatively had limited recovery from sepsis. He was scheduled two weeks later for window thoracostomy and vac-therapy. At re-thoracotomy, the thickened parietal pleura was easily mobilized from the chest wall; therefore, the intercostal muscles were carefully dissected off the ribs and, along with the thick pleura, were placed deep in the pleural cavity and sutured at the right hemidiaphragm and lower lobe visceral pleura, using prolene 3-0 sutures. Two chest drains were inserted, and a vacuum device was applied in the subcutaneous space, rather than a drain, because the skin and subcutaneous layers were inflamed from the thoracotomy for middle-lobectomy that had been performed 4 weeks earlier. The patient recovered from pleural sepsis with vacuum therapy discontinued after 4 weeks with good wound healing. He remained afebrile and hemodynamically stable without any supplemental oxygen, and free from a demanding thoracostomy window that would require months-long attendance by specialist nurses.

### 3.3. Patient 3

**Figure 4:**
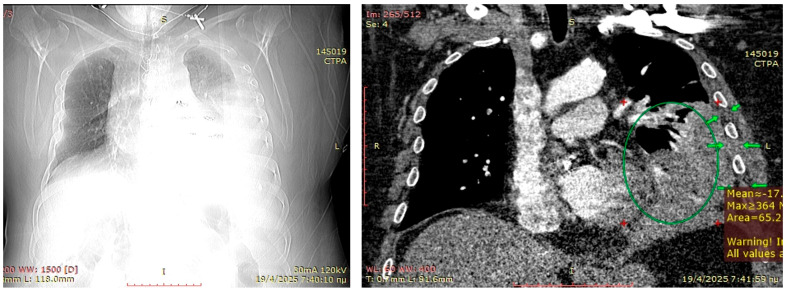
Preoperative planning CT-scan. Although the topogram similar to a standard antero-posterior chest x-ray shows the approximate extent of a parapneumonic effusion and an estimation of the location, it is of little practical value compared to a CT-scan. The right coronal view of the CT-scan is of much higher value to recognize the severe parenchymal infiltration (green circle) which prohibits decortication and describes the thickness of the chest wall to estimate if a Sawamura thoracoplasty by soft-tissue mobilization can be attained (green arrows).

A 55-year-old male obese (BMI > 35) smoker developed pneumonia, which was partially treated with successive antibiotic courses, eventually leading to antibiotic resistance, and a parapneumonic pleural effusion treated with an ultrasound guided small-bore Seldinger-type pleural catheter. Upon spending three weeks in the respiratory ward, he developed hemoptysis while receiving low-molecular-weight heparin for treatment of segmental pulmonary emboli. The hemoptysis decreased at 2 days after heparin cessation and tranexamic acid administration; however, his respiratory physiology remained suboptimal, raising concerns either for pulmonary embolism or parenchymal necrosis. A CT pulmonary angiogram performed for excluding new pulmonary embolism revealed left lower lobe necrosis with contrast leakage in the pleural cavity (Figure 4). A small ruptured abscess could not be excluded. On acute basis, the patient underwent an exploratory left lower antero-lateral thoracotomy at the 7th intercostal space. The pleural cavity had blood clots present, and the lower lobe was necrotic on the lateral segment. Selective bronchial closure for single lung ventilation was insufficient to maintain acceptable O_2_ saturations, precluding our initial plan for a salvage lower lobectomy. A wide-wedge resection was performed on the necrotic lung, and the long stapling line was reinforced with a Serratus anterior flap. Decortication was partially performed with eventual suboptimal lung expansion. To anticipate a chronic infected space, a muscular ‘blanket’ of the intercostal muscles from ribs 5–8 and the underlying thick parietal pleura was prepared and with the serratus anterior flap was secured in the cavity. Two chest drains were inserted, and a smaller bore drain was inserted in the subcutaneous space. The patient recovered from pleural sepsis, regaining almost complete lung expansion. Within 45 days, he achieved full recovery and returned to his previous physically demanding occupation.

## 4. Results

The mean age of our patients was 63.3 years. The mean operative time was 170 min, and the average blood loss was 425 mL. No re-operations were necessary, and no infectious complications occurred, indicating the safety and efficacy of this procedure. All patients received postoperatively culture-driven antibiotics without the need for conversion. Upon completion of antibiotic courses, none became febrile or septic, indicating complete source control by surgery. All patients were admitted postoperatively to the ICU for an average of 5 days. Follow-up CXR were enough to exclude clinically significant residual spaces or abscesses, and to assess lung re-expansion. All patients received intravenous analgesia by a combination of tramadol and paracetamol as per thoracotomy for cancer for one week and thereafter on a per os combination for two more weeks. No patient received analgesia after the third week or complained of chronic pain or upper-body dismobility.

## 5. Discussion

For decades now, several operative techniques have been developed for managing complex postinfection or postoperative empyema unresponsive to standard medical or interventional treatment(s). An established option is chest fenestration or Eloesser flap, which allows the inspection and debridement of the septic pleural cavity, controls infection, improves the patient’s general condition, and facilitates discharge into community care, if available, for daily cavity irrigation [12]. As standard, the patient requires daily dressing changes or nowadays the use of vacuum-assisted devices until the cavity is sterile and often spontaneously obliterated. However, a second procedure for muscle flap transfer is required in most cases to restore the normal appearance once infection control is attained [13,14]. This method can be successful in compliant patients with direct access to a community care team with expertise in chronic wound management. This entails a substantial cost to the healthcare system and social inconvenience with a grossly disfiguring and unacceptable cosmetic outcome. At some institutions, an initial fenestration is fast-tracked within the same admission, with frequent cavity washings and dressing changes performed until the cavity is sterile and ready for secondary closure using either a Clagett procedure or muscle flap transfers. While these methods are best suited in tertiary centers with substantial budgets, it is not applicable in health systems with deficiencies of financial resources, staff, bed capacity, and patient compliance [15]. Moreover the abovementioned procedures require a resection of the ribs which results in chest deformation, impairs postoperative lung function and causes postural deformity not appreciated by patients of any age [16,17].

Here we described the use of a modified Sawamura technique (also known as Kinchu method or extraperiosteal thoracoplasty) in three patients [18,19]. Although this method is largely disused for unknown reasons, it represents a suitable choice for practices with staff shortages and settings where patient compliance is an issue. The obvious advantage of this thoracoplasty is the single-stage treatment with the lowest possible clinical cost and reduced regular visits or repeated hospitalizations. Ultimately, postoperative care is continued in general practice or at home. Notably, the Sawamura technique yields good cosmetic results, and is well-tolerated by patients [17,20]. Sawamura thoracoplasty can be utilized in the same group of patients who qualify for the Eloesser procedure or full thoracoplasty but the decision for Sawamura thoracoplasty depends on the surgeons’ experience, the surgical department resources, and the quality of community care. As expected, there are no comparative studies between this technique and other forms of thoracoplasty. This technique remains in the shadow of the literature, and it would take significant time to be adopted in numbers that permit comparative studies in large cohorts. Our study is limited by low numbers of patients as most patients are treated properly in multidisciplinary settings.

The first study on the Sawamura technique by Iioka et al. demonstrated promising results as it was successful in 60 out of 65 patients and significantly improved pulmonary function measured by vital capacity (VC) and forced expiratory volume in 1 s (FEV1) [17]. Nakaoka and colleagues further evaluated the efficacy of Sawamura thoracoplasty in a small group of patients (*n* = 11), which did not respond to simple decortication. The procedure was successful in all patients and resulted in the re-expansion of the lung and relief in symptoms. There was however no significant improvement in %VC and %FEV1, which was probably a result of poor pulmonary function prior to the operation in the group of patients qualified for the Sawamura thoracoplasty [19]. Interestingly, the use of Sawamura thoracoplasty (as a part of a larger study) was also reported in a pediatric patient; however, there are no exact details on the outcome [21]. Other reports of the use of Sawamura thoracoplasty are rare rather scarce [22,23].

**Figure 5:**
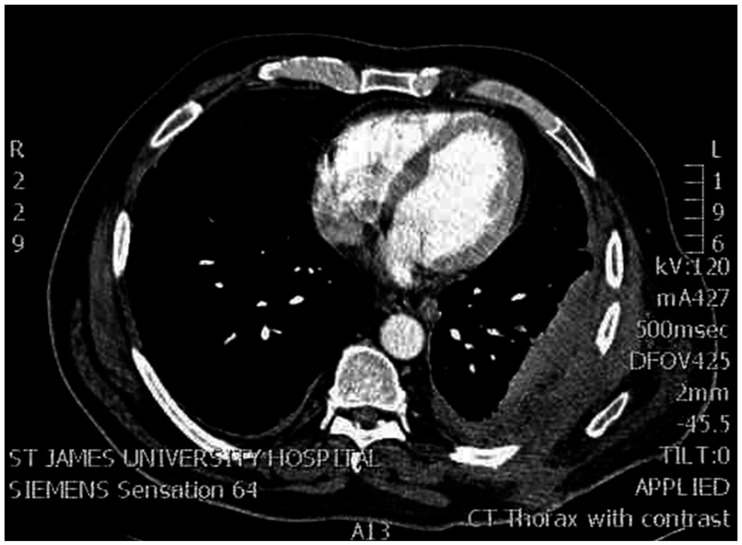
A post-operative CT scan from the archive of our clinic showing complete obliteration of the pleural space and opposition of soft chest wall elements with the partially collapsed left lower lobe.

Regarding surgical planning and decision making, a CT scan is mandatory as is required for all thoracoplastic procedures. The thoracotomy incision should correlate to the location of the infected pleural space. Thus, we should be ready to tailor our approach into atypical thoracotomies. The large muscles of the thorax—latissimus dorsi and serratus anterior—should ideally be preserved for flap use if required as in two of our patients. Gentle ventilation is used to define the volume and position of this space, and to identify the exact position of the thoracoplastic ‘patch’. During the procedure, periosteal rib stripping should be generous enough to comfortably fill the cavity without tension. The integrity of the intercostal neurovascular bundles should be respected to prevent tissue ischemia. All soft tissues of the chest wall should be preserved as a single sheet, and the thoracotomy incision sealed without any extra-pleural communication. Moreover, this space must be adequately drained to avoid hematoma formation. Moderate parenchymal leaks should no longer be of concern as long as the collapsed chest wall elements cover the lung parenchyma. After the operation, the extraperiosteal space fills with exudate which exerts pressure on the collapsed chest wall elements facilitating the obliteration of the cavity of empyema. In the following months, the exudate is absorbed as the lung re-expands [17,18]. Figure 5. shows a CT scan of a patient after Sawamura thoracoplasty with obliterated pleural space. At the end of the procedure, at least one intrapleural and one extrapleural chest drain are required. We recommend applying suction on both drains, until drainage is diminished and the lung expands on chest films. Patients should undergo early postoperative mobilization, aggressive pulmonary expectoration, shoulder mobilization, adequate pain management by combination of analgesics and nutritional support. Antibiotic treatment is culture-guided by the intraoperative obtained samples, especially if multi-resistant strains have been isolated.

All our patients recovered well and despite the limited number of our cases, we believe that the Sawamura technique appears to have significant benefits. Although it is classified as a thoracoplasty, the mechanical properties of the thoracic cage are retained (similarly to the Eloesser procedure), ensuring normal ventilation. Moreover, shoulder joint function is not affected, and patients are left with no visible or palpable deformity, which has a positive psychological impact. In cases where severe rib crowding prevents access to the pleural cavity, we advise cutting but not removing the proximal and distal rib. Importantly, with this method, there is no need to re-visit the pleural cavity, thereby reducing the overall treatment cost and avoiding schedule barriers in busy general hospitals. Another important advantage of the modified Sawamura thoracoplasty is the lack of community care requirements. These benefits make this procedure attractive for practices with limited resources, patients with low compliance, or high-risk individuals under anticoagulation (like patient 1 in our series).

## 6. Conclusions

We conclude that rib-sparing thoracoplasty by Sawamura technique is suitable for patients with complex chronic pleural empyema as an effective alternative to long-term pleural drainage and/or deforming fenestration and is ideal when patient compliance is an issue. More exposure of this technique through scientific meetings and interactive media should facilitate the acknowledgement of the method.

## Data Availability

The data presented in this study are available in this article.

## Abbreviations

The following abbreviations are used in this manuscript:

VATS	Video-assisted thoracoscopic surgery
HDU	High-dependency unit
ICU	Intensive care unit
CT	Computed tomography
VC	Vital capacity
FEV1	Forced expiratory volume in 1 s
